# Electronic screening as a design lever in La-doped BaTiO_3_: coupling between polarization, dielectric response, and optical anisotropy

**DOI:** 10.1039/d6ra08623c

**Published:** 2026-10-26

**Authors:** Sikander Azam, Muhammad Fareed, Qaiser Rafiq, Jan Minar, Abed Alataway

**Affiliations:** a University of West Bohemia in Pilsen, New Technologies – Research Centre 8 Univerzitní Pilsen 306 14 Czech Republic azam@ntc.zcu.cz; b Faculty of Engineering and Applied Sciences, Department of Physics, RIPHAH International University Islamabad Pakistan; c School of Physics and Optoelectronic Engineering, Beijing University of Technology Beijing 100124 China; d Prince Sultan Institute for Environmental, Water and Desert Research, King Saud University Riyadh 11451 Saudi Arabia

## Abstract

Donor substitution is an effective strategy for modifying the coupled ferroelectric, dielectric, electronic, and optical properties of perovskite oxides by altering lattice distortion and electronic screening. In this work, first-principles calculations were performed to examine the microscopic effect of La donor substitution on BaTiO_3_. A representative La-doped model, corresponding to 12.5% substitution at the Ba site, was investigated using density functional theory, density-functional perturbation theory, and the modern Berry-phase formalism. The reported lattice parameters indicate anisotropic contraction and a slight increase in tetragonality upon La incorporation, while the reported internal displacements and relative phase energies indicate decreased Ti off-centering and reduced energetic stabilization of the tetragonal structure relative to the cubic reference. The Berry-phase polarization is reduced accordingly, mainly due to enhanced donor-induced electronic screening and a decrease in anomalous dynamical charge transfer. Phonon dispersion calculations confirm that the La-doped structure remains dynamically stable, while the hardening of the low-frequency polar mode indicates suppression of the ferroelectric soft-mode instability. The dielectric response further reflects this behavior: the high-frequency dielectric constant increases moderately due to enhanced electronic screening, whereas the static dielectric response decreases as the lattice-mediated contribution is weakened. Electronic-structure calculations show that La doping shifts the Fermi level toward the conduction band without generating mid-gap defect states, leading to a Burstein–Moss-type shift of the optical absorption edge. The optical spectra also indicate improved near-edge transparency and reduced birefringence. The latter accompanies lower spontaneous polarization, although the reported lattice constants do not support attributing it to decreased tetragonality. Overall, the findings suggest a consistent microscopic connection between La-induced electronic screening, soft-mode modifications, reduced anomalous Born effective charges, polarization suppression, and decreased optical anisotropy. Since only one La concentration is explicitly considered, the 12.5% model is treated as a representative moderate-doping case rather than a complete concentration-dependent description. The results provide mechanistic insight into how donor substitution can tune the coupled ferroelectric and optical response of BaTiO_3_-based oxides, while further calculations at multiple dopant concentrations would be valuable for establishing full quantitative doping trends.

## Abstract 2

La substitution in BaTiO_3_ modifies electronic screening and Ti off-centering, linking reduced ferroelectric polarization with changes in dielectric response and optical anisotropy.

## Graphical abstract

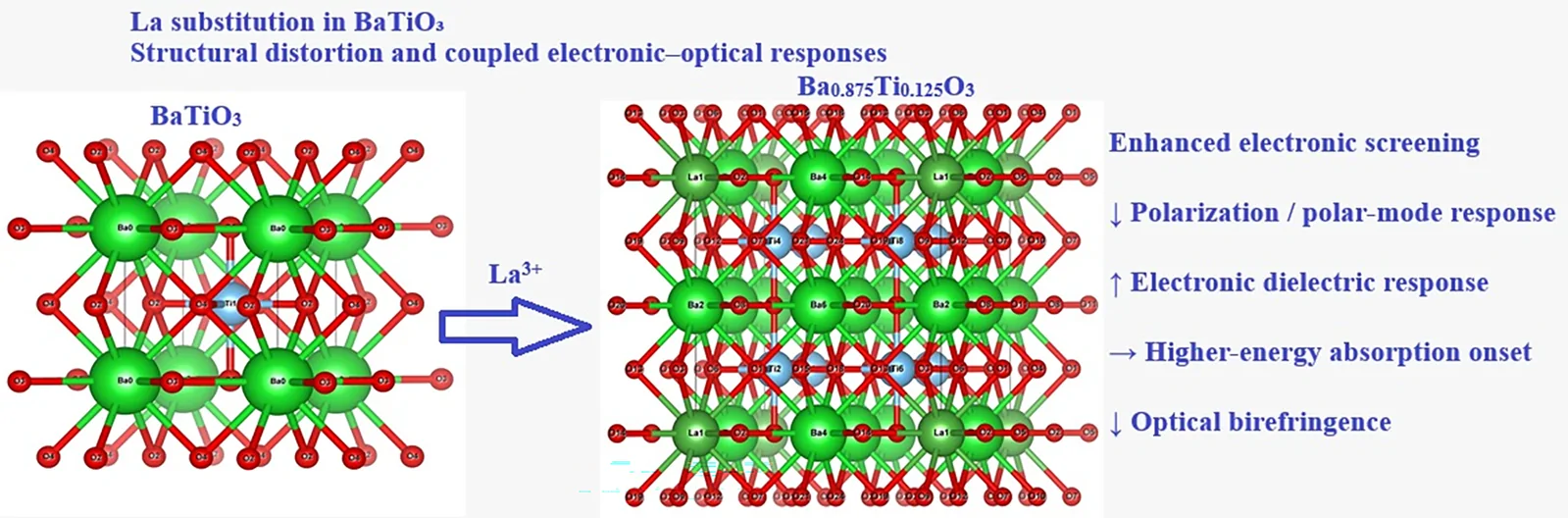

## 1. Introduction

Ferroelectric perovskite oxides occupy a central position in functional materials research because of their strong coupling between lattice distortion, charge redistribution, polarization, dielectric response, and optical behaviour. Among them, barium titanate (BaTiO_3_) is a prototypical displacive ferroelectric and has long served as a model system for understanding phase transitions, polarization mechanisms, and dielectric anomalies in ABO_3_ perovskites.^1–3^ The tetragonal ferroelectric phase of BaTiO_3_ originates from a delicate balance between short-range repulsive forces and long-range Coulomb interactions, stabilized by Ti–O hybridization and anomalously large Born effective charges.^4,5^ This sensitivity of the polar ground state to lattice distortion and electronic screening makes BaTiO_3_ highly responsive to chemical substitution.

Chemical substitution in BaTiO_3_ is commonly classified into donor and acceptor doping, and these two routes modify the functional response in different ways. Acceptor dopants usually promote charge compensation through oxygen vacancies or defect complexes, which can pin domain walls, generate internal bias fields, and produce hard-ferroelectric behaviour. In contrast, donor dopants, such as trivalent rare-earth ions at the Ba^2+^ site or pentavalent ions at the Ti^4+^ site, introduce excess electronic charge and enhance screening of the long-range dipole–dipole interactions responsible for ferroelectric instability.^6–11^ As a result, donor doping generally reduces tetragonality, Ti off-centering, spontaneous polarization, and ferroelectric hysteresis while often maintaining a large dielectric response.^6–11^

Among donor dopants, La^3+^ substitution at the Ba^2+^ site has been widely used to tune the electrical and dielectric properties of BaTiO_3_.^6–8^ Replacing Ba^2+^ with La^3+^ introduces donor electrons, modifies the lattice through chemical pressure, and weakens the ferroelectric distortion by enhancing electronic screening. Experimentally, La-doped BaTiO_3_ is known to show reduced tetragonal distortion, suppressed spontaneous polarization, lower Curie temperature, and modified dielectric behaviour.^9–11^ These properties have motivated its use in multilayer ceramic capacitors, thermistors, and tunable dielectric components.

Although the present work focuses on La substitution, the underlying donor-screening mechanism is relevant to a broader class of donor-doped BaTiO_3_ systems. Representative donor dopants include rare-earth ions such as Ce, Nd, Sm, and Gd at the Ba site, as well as Nb and Ta at the Ti site. Their qualitative influence on ferroelectricity is often similar, but the quantitative response depends on ionic size, valence state, local bonding environment, defect-compensation mechanism, and the possible presence of localized f or d states. La^3+^ is selected here as a chemically clean A-site donor prototype because it readily substitutes for Ba^2+^, donates electronic charge, and does not introduce partially occupied magnetic 4f states near the band edges. The expected roles of representative donor dopants in BaTiO_3_ are summarized in Table 1.

**Table 1:** Comparative role of representative donor dopants in BaTiO_3_ and their expected influence on ferroelectric behavior. La^3+^ is treated in the present work as a chemically clean A-site donor prototype, while Ce and other rare-earth or B-site donor dopants may introduce additional dopant-specific effects such as mixed valence, localized f states, or direct modification of the Ti–O electronic network

Dopant type	Expected role in BaTiO_3_	Possible effect on ferroelectricity	Dopant-specific issue
La^3+^ at Ba^2+^ site	Clean A-site donor	Reduces tetragonality, Ti off-centering, and polarization through electronic screening	No partially occupied 4f states near band edges
Ce^3+^/Ce^4+^	Donor or mixed-valence dopant	May suppress polarization, but effect depends on charge state	Mixed valence and possible defect compensation
Nd^3+^/Sm^3+^/Gd^3+^	Rare-earth donor dopants	Similar donor-screening trend expected	Local 4f states may affect optical or magnetic response
Nb^5+^/Ta^5+^ at Ti^4+^ site	B-site donor dopants	Strong carrier-induced screening; may affect Ti–O network directly	Alters B-site electronic structure more directly than La

Methodologically, first-principles descriptions of ferroelectric perovskites rely on density-functional perturbation theory for phonons, Born effective charges, and dielectric tensors,^12,13^ the modern Berry-phase theory for spontaneous polarization,^14^ and established formalisms for optical and electro-optical response.^15,16^ These coupled electromechanical properties are also relevant to the broader use of piezoelectric thin films in microelectromechanical systems.^17^

From a microscopic perspective, the response of BaTiO_3_ to donor doping involves several interconnected effects. Excess electronic charge screens the long-range Coulomb interactions that stabilize the ferroelectric soft mode, while A-site substitution modifies lattice anisotropy and Ti off-centering. In addition to affecting ferroelectric and dielectric behaviour, donor doping can also modify the optical response by shifting the Fermi level toward the conduction-band minimum and producing a Burstein–Moss-like displacement of the optical absorption edge.^18,19^ At the same time, reduced tetragonality and polarization anisotropy are expected to decrease optical birefringence. Therefore, a complete description of La-doped BaTiO_3_ requires structural, ferroelectric, dielectric, electronic, phonon, and optical properties to be considered together.

Despite extensive experimental and theoretical work on donor-doped BaTiO_3_, many previous studies have focused on selected aspects of the doping response, such as lattice distortion, dielectric permittivity, electronic band modification, carrier-induced screening, or optical absorption. The individual trends of polarization reduction, soft-mode hardening, and Burstein–Moss-type optical-edge shifts are already known in donor-doped BaTiO_3_ systems. However, the direct atomistic connection between these effects remains less clearly established. In particular, the relationship between La-induced electronic screening, reduced Ti off-centering, weakened anomalous Born effective charges, Berry-phase polarization suppression, modified dielectric response, and reduced optical birefringence has not been systematically examined within one internally consistent first-principles framework.

To clarify the specific contribution of the present work relative to earlier donor-doped BaTiO_3_ studies, a comparison is provided in Table 2. The novelty of this study lies not in treating La substitution itself as a new phenomenon, but in connecting the structural, ferroelectric, dielectric, electronic, and optical responses through a common microscopic mechanism.

**Table 2:** Comparison of common themes in previous studies with the scope of the present work

Aspect	Common focus in previous studies	Added value of the present work
Structural distortion	Changes in lattice parameters and tetragonality under donor doping	Comparison of lattice contraction with internal polar displacements, distinguishing volume changes from changes in tetragonality
Ferroelectric polarization	Changes in spontaneous polarization with donor concentration	Berry-phase comparison of pristine and La-substituted structures, considered alongside Ti off-centering and Born effective charges
Phonon behavior	Polar-mode suppression or hardening in doped or charged BaTiO_3_	Comparison of reported polar-mode hardening with internal displacements and tetragonal stabilization energy
Dielectric response	Changes in static and high-frequency dielectric constants	Separate assessment of electronic and lattice-mediated dielectric contributions
Electronic structure	Fermi-level shifts and donor-induced n-type character	Examination of near-band-edge electronic changes and the absence of mid-gap states in the investigated La-substituted model
Optical response	Burstein–Moss shifts, absorption-edge changes, and refractive-index variations	Comparison of reduced birefringence with polarization and internal displacement changes, without attributing it to decreased tetragonality
Overall contribution	Emphasis on selected structural or functional properties	Combined assessment of structural, energetic, polar, dielectric, and optical responses for pristine BaTiO_3_ and one representative La-substituted configuration

In this work, we present a first-principles study of La-doped BaTiO_3_ using density functional theory, density-functional perturbation theory, phonon analysis, and the Berry-phase formalism. A representative Ba_0.875_La_0.125_TiO_3_ model is used to examine how La donor substitution modifies lattice parameters, tetragonality, Ti off-centering, phonon stability, Born effective charges, spontaneous polarization, dielectric response, electronic structure, and optical anisotropy. The 12.5% La concentration is treated as a representative moderate-doping case rather than a complete concentration-dependent description. The objective is to identify how donor-induced electronic screening links the suppression of ferroelectric lattice instability with changes in dielectric and optical properties. This integrated analysis provides a mechanistic basis for understanding donor-engineered BaTiO_3_ and related ferroelectric oxides while avoiding overgeneralization beyond the specific doping model considered here.

## 2. Computational methodology

All first-principles calculations were carried out within density functional theory (DFT) using a plane-wave pseudopotential formalism.^20,21^ Exchange–correlation effects were treated using the Perdew–Burke–Ernzerhof functional revised for solids (PBEsol).^22,23^ Core–valence interactions were described using projector augmented-wave (PAW) potentials, with Ba (5s^2^5p^6^6s^2^), La (5s^2^5p^6^5d^1^6s^2^), Ti (3p^6^3d^2^4s^2^), and O (2s^2^2p^4^) treated explicitly as valence electrons.

La substitution was modeled using a (2 × 2 × 2) supercell of the five-atom perovskite cell, containing 40 atoms. One Ba atom was replaced by La, giving Ba_0.875_La_0.125_TiO_3_ and 12.5% substitution on the Ba sublattice (see Fig. 1). This model represents a periodic arrangement of substitutional La atoms. Structural relaxation was performed in a non-magnetic configuration, and subsequent spin-polarized electronic calculations were used to examine possible magnetic symmetry breaking.

**Fig. 1:**
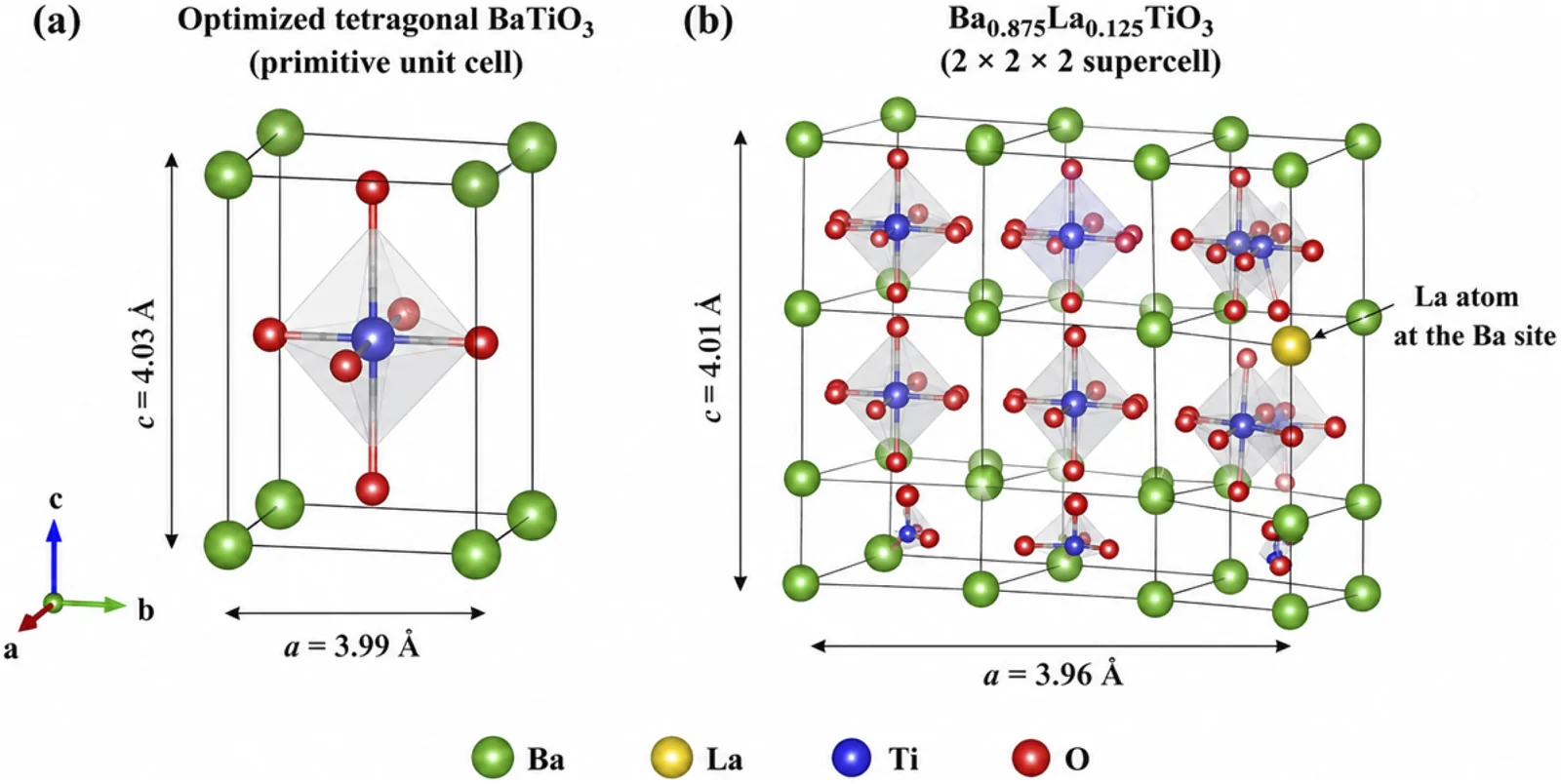
Optimized crystal structures of (a) tetragonal BaTiO_3_ primitive unit cell and (b) Ba_0.875_La_0.125_TiO_3_ (2 × 2 × 2) supercell with one La atom substituting a Ba site (12.5% La). The lattice parameters reported for these structures are listed in Table 3. Color code: Ba (green), La (yellow), Ti (blue), and O (red).

Both lattice parameters and internal atomic coordinates were relaxed until residual Hellmann–Feynman forces were below 10^−3^ eV Å^−1^ and the total-energy change between successive ionic steps was below 10^−6^ eV. A plane-wave kinetic-energy cutoff of 500 eV was used. Brillouin-zone integrations employed Monkhorst–Pack *k*-point meshes of (6 × 6 × 6) for the primitive cell and (3 × 3 × 3) for the supercell; these settings were verified to yield converged structural and electronic properties.

Ferroelectric polarization was evaluated using the modern Berry-phase formalism.^14,24^ The spontaneous polarization, *P*_s_, was obtained from the polarization difference between the relaxed tetragonal structure and a centrosymmetric reference constructed by symmetrizing the atomic positions while preserving the lattice parameters. The validity condition for this calculation is stated in Section 2.1.

Dielectric properties were evaluated within density-functional perturbation theory (DFPT).^12,13^ The high-frequency electronic dielectric tensor, *ε*_∞_, was obtained from linear response to a macroscopic electric field. The lattice contribution was calculated from zone-center phonon modes and Born effective charges, and the static dielectric response was constructed as *ε*_0_ = *ε*_∞_ + *ε*_ionic_. Born effective charge tensors were calculated to assess the coupling between atomic displacements and macroscopic polarization.

Electronic band structures and densities of states (DOS) were calculated for the relaxed geometries using the *k*-point meshes specified above.^28^ Spin-polarized calculations yielded negligible spin polarization for the investigated La-substituted model, with nearly identical spin-up and spin-down electronic structures. The Fermi-level position was determined self-consistently to examine donor-induced changes in electronic occupations and near-band-edge states.

Optical properties were evaluated from the frequency-dependent complex dielectric function, *ε* (*ω*) = *ε*_1_ (*ω*) + i*ε*_2_ (*ω*), within the independent-particle approximation using interband transition matrix elements and the PAW-based optical formalism.^25^ Absorption coefficients, refractive indices, and birefringence were derived using standard optical relations.^15^ Pristine and La-substituted structures were compared using identical computational settings. A possible Burstein–Moss-type displacement of the absorption onset was examined by comparing *ε*_2_ (*ω*) with the reported electronic occupations and Fermi-level movement.

### 2.1. Scope and limitations

The calculations compare pristine BaTiO_3_ with one periodic Ba_0.875_La_0.125_TiO_3_ configuration. They do not establish concentration-dependent scaling or account for alternative dopant arrangements and charge-compensation mechanisms.

Semilocal DFT generally underestimates semiconductor band gaps. Accordingly, the calculated optical energies are approximate, and the discussion emphasizes comparisons obtained using identical computational settings. Quantitative agreement of the doping-induced shifts with experiment is not established. The independent-particle optical treatment also omits excitonic and quasiparticle corrections.

Conventional Berry-phase polarization requires an insulating occupied manifold throughout the path connecting the reference and polar structures.^26,27^ The reported polarization values are therefore conditional on this requirement being satisfied. A gap between the valence and conduction bands, or the absence of mid-gap impurity states, alone does not establish applicability if conduction bands are partially occupied.

## 3. Results and discussion

### 3.1. Structural properties

#### 3.1.1. Lattice parameters and tetragonality

BaTiO_3_ crystallizes in a tetragonal perovskite structure at room temperature, characterized by a slight elongation along the crystallographic *c* axis that reflects the underlying ferroelectric distortion. Within PBEsol, the reported optimized lattice parameters of pristine BaTiO_3_ are *a* ≈ 3.99 Å and *c* ≈ 4.03 Å. These displayed values give *c*/*a* ≈ 1.0100 and a unit-cell volume of approximately 64.16 Å^3^, calculated using *V* = *a*^2^*c*. These values are in close agreement with experimental room-temperature data (*a* ≈ 3.994 Å, *c* ≈ 4.038 Å) and prior first-principles studies employing PBEsol or LDA-based approaches, which are known to reproduce ferroelectric distortions in BaTiO_3_ with good fidelity.

For the representative 12.5% La-substituted model, the reported lattice parameters are *a* ≈ 3.96 Å and *c* ≈ 4.01 Å. These values give a unit-cell volume of approximately 62.88 Å^3^, corresponding to a contraction of approximately 1.99% relative to the pristine value of 64.16 Å^3^. The relative contractions along a and c are approximately 0.75% and 0.50%, respectively. Consequently, the reported lattice constants indicate anisotropic contraction accompanied by a small increase in *c*/*a*, from approximately 1.0100 to 1.0126.

The lattice contraction should be distinguished from the change in tetragonality: a smaller unit-cell volume does not necessarily imply a lower *c*/*a* ratio. Although the reported lattice constants indicate contraction upon La substitution, they yield a slight increase in *c*/*a*. Therefore, these structural values alone do not establish suppression of the tetragonal distortion. The reported changes in Ti off-centering, polarization, and phase stabilization energy are considered separately in the following sections.

#### 3.1.2. *c/a* ratio and tetragonal distortion

Tetragonality is conventionally quantified by the *c*/*a* ratio, which measures the axial distortion of the tetragonal lattice. For pristine BaTiO_3_, the reported lattice parameters give *c*/*a* ≈ 1.0100, close to the experimental value of approximately 1.0110 listed in Table 3. The deviation from unity indicates an elongation along the c axis. However, this lattice metric alone does not quantify the internal polar displacements or spontaneous polarization.

**Table 3:** Calculated structural parameters and phase stability of pristine and La-doped BaTiO_3_. Here, *a* and *c* are the optimized lattice parameters, *c/a* is the tetragonality ratio, *V* is the unit-cell volume, and Δ*E* = *E*_cubic_ − *E*_tetragonal_ represents the energetic stabilization of the tetragonal phase. Positive Δ*E* indicates that the tetragonal ferroelectric phase is more stable than the cubic phase. The *c*/*a* ratios and volumes were calculated from the lattice parameters displayed in the table using *c*/*a* and *V* = *a*^2^c

System	*a* (Å)	*c* (Å)	*c*/*a*	*V* (Å^3^)	Δ*E* (meV per f.u.)
BaTiO_3_ (DFT, PBEsol)	3.99	4.03	1.0100	64.16	42
BaTiO_3_ (Expt.)	3.994	4.038	1.0110	64.41	
Ba_0.875_La_0.125_TiO_3_ (DFT)	3.96	4.01	1.0126	62.88	23

For the 12.5% La-substituted model, the reported lattice parameters give *c*/*a* ≈ 1.0126, compared with approximately 1.0100 for pristine BaTiO_3_. Thus, Table 3 indicates a small increase in tetragonality rather than the previously stated decrease. Separately, the reported energy difference between the cubic and tetragonal structures decreases from approximately 42 to 23 meV per f.u. This smaller energy difference indicates reduced energetic stabilization of the tetragonal phase, but it cannot be attributed to a reduction in *c*/*a* on the basis of the reported lattice parameters.

La substitution can influence polar distortion through changes in the local bonding environment and electronic screening. These effects must be assessed using internal atomic displacements, electronic response, and relative phase energies, rather than inferred from lattice contraction alone. In the present results, the reported decrease in tetragonal stabilization energy and Ti off-centering should therefore be distinguished from the slight increase in the axial ratio derived from Table 3.

Table 3 shows calculated lattice parameters (*a*, *c*), tetragonality (*c/a*), unit-cell volume (*V*), and total-energy difference between the tetragonal and cubic phases (Δ*E* = *E*_cubic_*– E*_tetragonal_) for pristine and La-doped BaTiO_3_. Experimental room-temperature values for pristine BaTiO_3_ are included for comparison. Positive Δ*E* indicates stabilization of the tetragonal ferroelectric phase.

The *c*/*a* ratios derived from the reported lattice constants remain above unity for both compositions, indicating that both cells retain a tetragonal metric. However, this metric alone does not establish ferroelectricity, which also requires assessment of the internal polar displacements and electronic state. Because the reported constants yield a slight increase in *c*/*a* upon La substitution, they do not demonstrate the reduction in tetragonality discussed in the earlier interpretation.

#### 3.1.3. Energetic stability of the tetragonal phase

To assess the relative stability of the tetragonal and cubic structures, the energy difference is defined as Δ*E* = *E*_cubic_ − *E*_tetragonal_. For pristine BaTiO_3_, the reported value is approximately 42 meV per formula unit, as listed in Table 3. This positive value indicates that the tetragonal structure is energetically favored over the cubic reference structure within the reported calculations.

For the 12.5% La-substituted model, the reported energy difference is approximately 23 meV per formula unit, compared with 42 meV per formula unit for pristine BaTiO_3_. The smaller positive value indicates a reduced energetic preference for the tetragonal structure relative to the cubic reference, while the tetragonal structure remains lower in energy among these two structures. This energetic change should be distinguished from the axial distortion: the lattice constants reported in Table 3 yield a slight increase in *c*/*a* and therefore do not support attributing the smaller energy difference to decreased tetragonality.

The smaller reported energy difference suggests that La substitution modifies the relative energetic preference for the tetragonal and cubic structures. However, this comparison alone does not determine the Curie temperature, dielectric response, or polarization hysteresis. Those properties require separate assessment, and the present energy values should be interpreted specifically as the relative energies of the two structures considered.

The reported structural parameters indicate that La substitution contracts the unit-cell volume while slightly increasing *c*/*a*. Separately, the reported energy difference between the cubic and tetragonal structures decreases from approximately 42 to 23 meV per formula unit. These results indicate that volume contraction, axial distortion, and relative phase stability do not change in parallel in the present comparison. Consequently, the reported suppression of polarization must be assessed using the internal atomic displacements and electronic response, rather than attributed to a decrease in tetragonality.

The reported reduction in spontaneous polarization upon La substitution accompanies a smaller energy difference between the cubic and tetragonal structures. However, the lattice constants in Table 3 yield a slight increase in *c*/*a*, so polarization suppression cannot be attributed to decreased tetragonality. Within the Berry-phase analysis, the reported polarization change should instead be examined alongside the internal polar displacements and anomalous Born effective charges. These quantities describe aspects of the polar response that are not determined by the axial ratio alone.

### 3.2. Dynamical stability: phonon dispersion analysis

The dynamical stability of pristine and La-doped BaTiO_3_ was examined through phonon dispersion calculations along high-symmetry directions of the Brillouin zone, as shown in Fig. 2. Phonon spectra provide a stringent criterion for structural stability, since imaginary (negative) frequencies indicate lattice instabilities that are not necessarily captured by total-energy minimization alone.

**Fig. 2:**
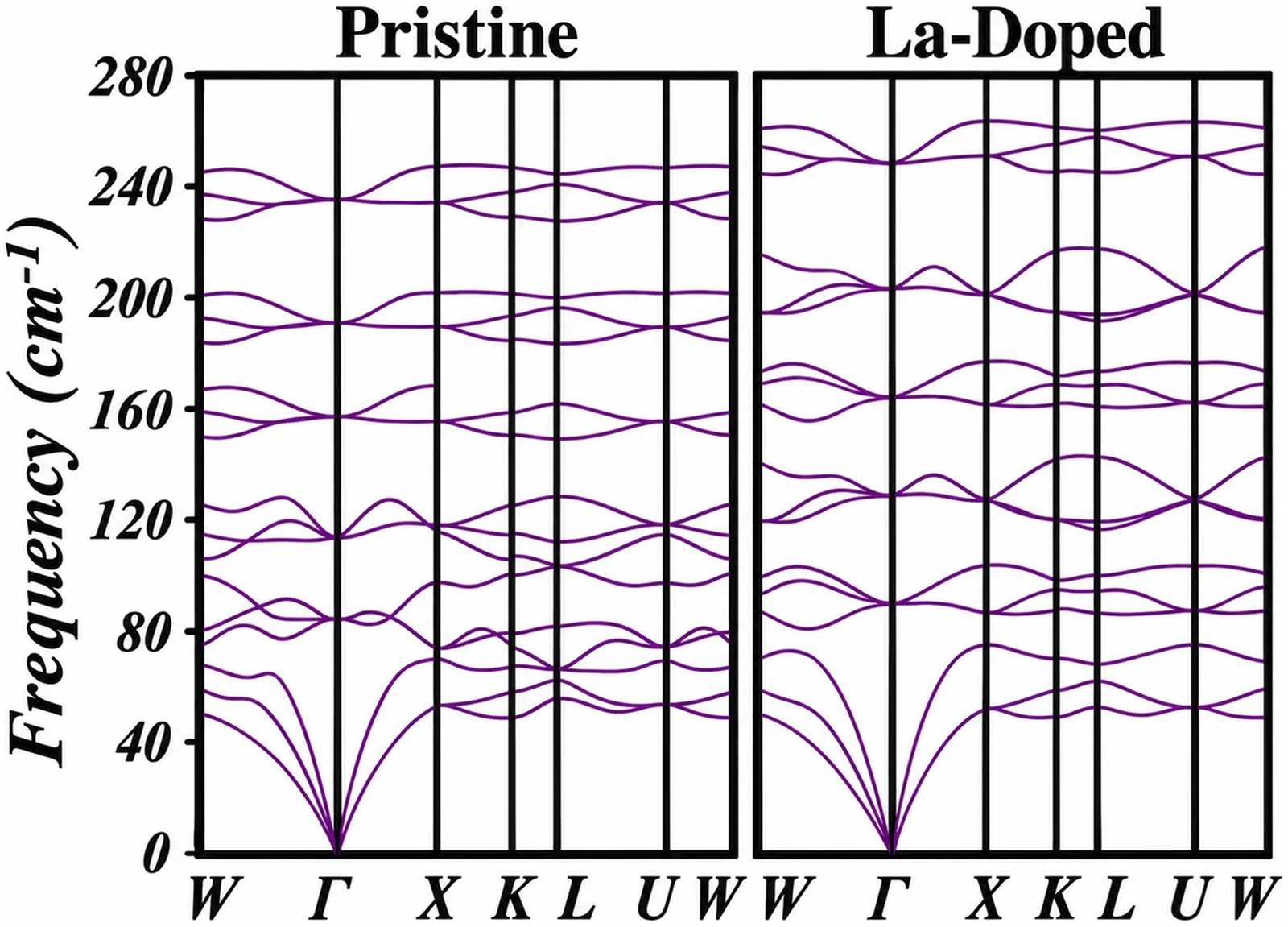
Calculated phonon dispersion relations of (a) pristine BaTiO_3_ and (b) La-doped BaTiO_3_ (12.5%) along high-symmetry directions of the Brillouin zone. The absence of imaginary frequencies confirms the dynamical stability of both structures. La substitution leads to a moderate hardening of low-frequency polar modes near the zone center, indicating suppression of the ferroelectric soft mode due to enhanced electronic screening, while preserving a stable tetragonal lattice.

For pristine tetragonal BaTiO_3_, the calculated phonon dispersion exhibits no imaginary frequencies throughout the Brillouin zone, confirming that the optimized ferroelectric structure corresponds to a dynamically stable ground state. The low-frequency transverse optical modes near the *Γ* point display pronounced softening, consistent with the well-established ferroelectric soft mode associated with Ti off-centering along the polar axis. This behavior reflects the proximity of BaTiO_3_ to the paraelectric phase and is characteristic of displacive ferroelectrics, in agreement with previous experimental and theoretical studies.

In the case of La-doped BaTiO_3_ (12.5%), the phonon spectrum likewise shows no imaginary frequencies along the entire W–Γ–X–K–L–U–W high-symmetry path, demonstrating that donor substitution does not induce any dynamical instability. Compared to the pristine compound, the low-frequency polar modes exhibit a clear upward shift in frequency, indicating a partial hardening of the ferroelectric soft mode. This frequency increase accompanies the reported reduction in Ti off-centering and may reflect changes in local bonding and electronic screening upon La substitution. It should not be attributed to decreased tetragonality, because the lattice parameters in Table 3 yield a slight increase in *c*/*a*.

Importantly, while the soft mode is weakened upon La substitution, it does not disappear entirely, indicating that the system remains in a dynamically stable polar state at this doping concentration. The absence of unstable acoustic or optical branches confirms that the reduced ferroelectric distortion originates from a genuine modification of the lattice energy landscape rather than from an incipient structural collapse or numerical artifact. These phonon results therefore provide a crucial consistency check for the structural and ferroelectric properties discussed above, establishing that La doping drives BaTiO_3_ toward the ferroelectric–paraelectric boundary while preserving overall vibrational stability.

### 3.3. Ferroelectric polarization

#### 3.3.1. Berry-phase polarization (*P*_s_)

Ferroelectricity in tetragonal BaTiO_3_ originates from a symmetry-breaking displacement pattern in which Ti shifts relative to the surrounding oxygen octahedron, accompanied by a correlated response of the O sublattice and the A-site framework. To quantify the resulting spontaneous polarization, we employed the modern theory of polarization (Berry-phase formalism), where P is obtained as a bulk geometric phase of the occupied Bloch manifold rather than a dipole moment of an arbitrarily defined unit cell.^29^ This approach is now standard for ferroelectric oxides and has been extensively applied to BaTiO_3_ and related perovskites.^30^ The calculated Berry-phase spontaneous polarization values, together with the corresponding Ti off-centering displacements and Born effective charges, are summarized in Table 4. More generally, strong coupling between polarization and lattice distortion is a well-established feature of perovskite ferroelectrics and underlies their pronounced electromechanical response.^31^ The polarization values in Table 4 are subject to the insulating-path requirement stated in Section 2.1.

**Table 4:** Calculated spontaneous polarization *P*_s_, Ti off-centering displacement along the *c* axis (Δ*z*_Ti_), and selected Born effective charges Z* for pristine and La-doped BaTiO_3_. Berry-phase polarization values are reported for the fully relaxed tetragonal structures. Experimental polarization values are shown for reference where available

System	*P*_s_ (µC cm^−2^)	Δ*z*_Ti_ (Å)	*Z*_Ti_^*^	*Z*_O∥_^*^
BaTiO_3_ (DFT, PBEsol)	25–27	0.12–0.14	+7.0 to +7.2	−5.6 to −5.8
BaTiO_3_ (experiment, 25 °C)^42^	∼26	—	—	—
Ba_0.875_La_0.125_TiO_3_ (DFT)	20–22	0.08–0.10	+6.6 to +6.8	−5.2 to −5.4

For pristine tetragonal BaTiO_3_, the calculated spontaneous polarization lies in the range *P*_s_ ≈ 25–27 µC cm^−2^, consistent with the established first-principles picture of BaTiO_3_ as a prototypical displacive ferroelectric stabilized by anomalous dynamical charge transfer and strong Ti–O hybridization.^30^ Upon La substitution at the Ba site (12.5% in a 2 × 2 × 2), Ps decreases from approximately 25–27 µC cm^−2^ to 20–22 µC cm^−2^, indicating a clear suppression of the polar distortion while retaining a finite polar ground state at this moderate donor concentration. The persistence of finite spontaneous polarization (*P*_s_) is consistent with the structural result that tetragonality remains above unity and the polar mode is weakened but not extinguished.

The reported decrease in spontaneous polarization upon La substitution accompanies decreases in Ti off-centering and the selected anomalous Born effective charges listed in Table 4. These changes suggest a modification of the internal polar response. However, they do not accompany decreased tetragonality: the lattice constants in Table 3 yield a slight increase in *c*/*a*. Moreover, comparison with a single doped composition does not establish a monotonic concentration dependence.

#### 3.3.2. Mechanism of polarization suppression: screening and reduced off-centering

The reduction of *P*_s_ under La doping can be understood as the combined consequence of (i) enhanced electronic screening associated with donor substitution and (ii) a geometric weakening of Ti off-centering driven by the modified A-site environment. Replacing Ba^2+^ with La^3+^ introduces extra electrons into the system; even when these carriers do not form a fully metallic state, the additional charge density increases the electronic polarizability and screens the long-range Coulomb interactions that stabilize the ferroelectric soft mode.^6–8^ In BaTiO_3_, the ferroelectric instability reflects a delicate balance between short-range repulsive forces and long-range Coulomb interactions, so increased donor-induced screening reduces the energetic gain associated with maintaining a large polar distortion. In practical terms, stronger screening therefore lowers the equilibrium amplitude of the polar mode and contributes to the reduction of spontaneous polarization.

La substitution also modifies the local A-site environment and internal atomic coordinates. The reported lattice constants indicate volume contraction accompanied by a slight increase in c/*a*; therefore, the reported decrease in Ti off-centering cannot be explained by reduced tetragonality. Instead, the internal displacement pattern must be considered separately from the axial ratio. Changes in Ti–O bonding and anomalous dynamical charge transfer provide relevant microscopic context for interpreting the polarization response,^32,33^ while previous work has examined the interplay between carrier density and polar distortion in BaTiO_3_.^34^

Overall, the reported Berry-phase polarization decreases upon La substitution, alongside smaller Ti off-centering and selected anomalous Born effective charges. Enhanced electronic screening provides a possible explanation for these changes. However, the reported lattice constants yield a slight increase in *c*/*a*, so the polarization reduction cannot be attributed to decreasing axial anisotropy. The comparison is restricted to the pristine structure and the single 12.5% La-substituted configuration investigated here.

### 3.4. Dielectric response

#### 3.4.1. Static and high-frequency dielectric constants (*ε*_0_ and *ε*∞)

The dielectric response of BaTiO_3_ is intrinsically tied to its ferroelectric lattice instability and anomalous dynamical charge transfer, consistent with the classical soft-mode description of ferroelectricity.^35,36^ Within density-functional perturbation theory (DFPT), the total static dielectric constant *ε*_0_ can be decomposed into an electronic (high-frequency) contribution *ε*∞ and a lattice-mediated ionic contribution, such that *ε*_0_ = *ε*_∞_ + *ε*_ionic_. Linear-response and DFPT formalisms provide a rigorous framework for evaluating these electronic and lattice contributions, as well as their coupling to atomic displacements and electric fields.^37,38^

For pristine tetragonal BaTiO_3_, the calculated high-frequency dielectric constant is *ε*_∞_ ≈ 5.3–5.6, in excellent agreement with experimental optical dielectric constants *ε*_∞_ ≈ 5.3–5.6 and prior first-principles studies.^39,40^ The static dielectric constant (*ε*_∞_ ≈ 5.4) is substantially larger (typically *ε*_∞_ ≈ 250–300 at 0 K in first-principles calculations), reflecting the strong contribution of the soft polar mode associated with Ti off-centering. This large lattice contribution is a hallmark of displacive ferroelectrics and underpins the exceptional dielectric and piezoelectric properties of BaTiO_3_.

Upon La substitution at the Ba site, *ε*∞ shows a modest increase, reaching values of approximately 5.6–5.9 at 12.5% La concentration. This enhancement originates from the increased electronic polarizability associated with donor electrons introduced by La^3+^ substitution, which enhance electronic screening without fundamentally altering the Ti–O bonding network. In contrast, the static dielectric constant *ε*_0_ exhibits a more nuanced behavior: while *ε*_lat_ is reduced due to the weakening of the ferroelectric soft mode, the increased electronic screening partially compensates for this reduction. As a result, *ε*_0_ remains large but less divergent than in pristine BaTiO_3_, consistent with experimental observations of donor-doped BaTiO_3_ exhibiting high dielectric permittivity with suppressed ferroelectric hysteresis.

#### 3.4.2. Born effective charges and microscopic origin of dielectric screening

The microscopic origin of the large dielectric response in BaTiO_3_ is rooted in anomalously large Born effective charges, which quantify the change in macroscopic polarization induced by atomic displacements. In pristine BaTiO_3_, the calculated Born effective charge of Ti is approximately +7.0 to +7.2, significantly exceeding the nominal ionic value of +4, while oxygen ions displaced parallel to the Ti–O bond exhibit values of approximately −5.6 to −5.8, consistent with previous first-principles analyses of anomalous dynamical charges in BaTiO_3_.^33,40^ These anomalous values arise from strong Ti–O covalency and dynamic charge transfer and play a central role in amplifying both the ferroelectric polarization and the lattice contribution to *ε*_0_. The close relationship between lattice dynamics, interatomic force constants, and phonon behaviour in BaTiO_3_ has also been established through first-principles phonon-dispersion calculations.^41^ La substitution leads to a systematic reduction of these anomalous dynamical charges. At 12.5% La concentration, *Z*_Ti_^*^decreases to approximately +6.6–6.8, while Z_O∥_^*^ is reduced to −5.2 to 5.4. The smaller reported Born effective charges accompany reduced Ti off-centering and may reflect changes in Ti–O bonding and electronic screening. They should not be attributed to reduced lattice anisotropy, because the lattice constants in Table 3 indicate a slight increase in *c*/*a*. From a dielectric perspective, smaller Z* values translate into weaker coupling between atomic displacements and macroscopic polarization, thereby reducing the lattice-mediated dielectric response.

Importantly, the decrease in Z* does not indicate a breakdown of the ferroelectric mechanism, but rather a controlled softening of the polar instability. The Ti–O network remains intact, and the dynamical charges remain substantially anomalous compared to purely ionic values, explaining why *ε*_0_ remains large despite the suppression of spontaneous polarization.

#### 3.4.3. Relationship between dielectric response, polarization, and structural distortion

The reported decrease in the lattice contribution to the static dielectric response accompanies smaller Ti off-centering, spontaneous polarization, and selected anomalous Born effective charges. Meanwhile, the increase in the high-frequency dielectric constant indicates enhanced electronic polarizability. These changes suggest modifications of both the electronic and lattice-mediated responses upon La substitution. However, they do not accompany decreased tetragonality: the lattice constants in Table 3 yield a slight increase in *c*/*a*. The internal polar response must therefore be distinguished from the macroscopic axial ratio.

Such behavior is technologically relevant for applications requiring large dielectric constants with minimized polarization hysteresis and leakage, such as multilayer ceramic capacitors and tunable dielectric components. From a fundamental standpoint, the present results reinforce the established picture of BaTiO_3_ as a soft-mode ferroelectric whose dielectric response can be continuously tuned through controlled modification of lattice instability and electronic screening.

#### 3.4.4. Benchmarking against experimental and previous theoretical data

Table 5 compares the reported properties with available experimental values and previously reported trends. For pristine BaTiO_3_, the listed lattice constants give *c*/*a* ≈ 1.0100, close to the experimental value of approximately 1.0110. The reported polarization and high-frequency dielectric response also provide comparisons with earlier work. However, agreement for the pristine compound does not independently validate the La-substituted structure. In particular, the doped lattice constants give *c*/*a* ≈ 1.0126 and therefore do not reproduce the reduction in tetragonality discussed in the earlier interpretation.

**Table 5:** Benchmarking of calculated properties against experimental and previous theoretical trends for pristine and La-doped BaTiO_3_

Property	Present calculation	Experimental/previous trend	Comment
Lattice parameters of pristine BaTiO_3_	*a* ≈ 3.99 Å, *c* ≈ 4.03 Å	*a* ≈ 3.994 Å, *c* ≈ 4.038 Å	Good agreement; validates structural optimization
*c*/*a* ratio of pristine BaTiO_3_	≈ 1.0100	≈ 1.011	Close agreement with the experimental axial ratio
*c*/*a* ratio of La-doped BaTiO_3_	≈ 1.0126	Reduced tetragonality reported in some La-doped systems	The listed lattice constants indicate a slight increase in *c*/*a*; the direction differs from the literature trend
Spontaneous polarization	∼26 to ∼21 µC cm^−2^ after La doping	Donor doping reduces polarization	Correct qualitative trend
High-frequency dielectric constant, *ε*∞	Pristine: ≈ 5.3–5.6; 12.5% La-substituted: ≈ 5.6–5.9	Typically around 5–6 for BaTiO_3_-based oxides	The two compositions are reported separately
Static dielectric response	Reduced lattice contribution after La doping	Donor doping suppresses soft-mode contribution	Trend consistent; exact values temperature-dependent
Optical absorption edge	Slight upward shift after La doping	Donor-induced Burstein–Moss-type shift reported in n-type oxides	Relative shift meaningful; absolute edge affected by DFT band-gap error
Fermi-level position	Moves toward/near CBM	La acts as donor and increases n-type carrier character	Consistent with donor substitution

For the La-substituted model, the reported decreases in spontaneous polarization and changes in electronic and optical response can be compared qualitatively with previous studies. Such comparisons must account for differences in composition, temperature, strain, and charge compensation. The lattice constants reported here indicate a slight increase in tetragonality, so agreement with the literature cannot be claimed for the direction of the *c*/*a* change. Consequently, the structural comparison does not independently validate the proposed screening mechanism.

Comparison with experiment must also account for temperature, grain size, domain-wall motion, strain, dopant distribution, and defect chemistry, which are not fully represented by the ideal periodic structures used here. The computational scope and electronic and optical limitations are summarized in Section 2.1.

### 3.5. Electronic structure

#### 3.5.1. Band structure of La-doped BaTiO_3_

The electronic band structure of pristine tetragonal BaTiO_3_ is characterized by an indirect band gap, with the valence-band maximum (VBM) derived primarily from O-2p states and the conduction-band minimum (CBM) dominated by Ti-3d states (see Fig. 3a and b).

**Fig. 3:**
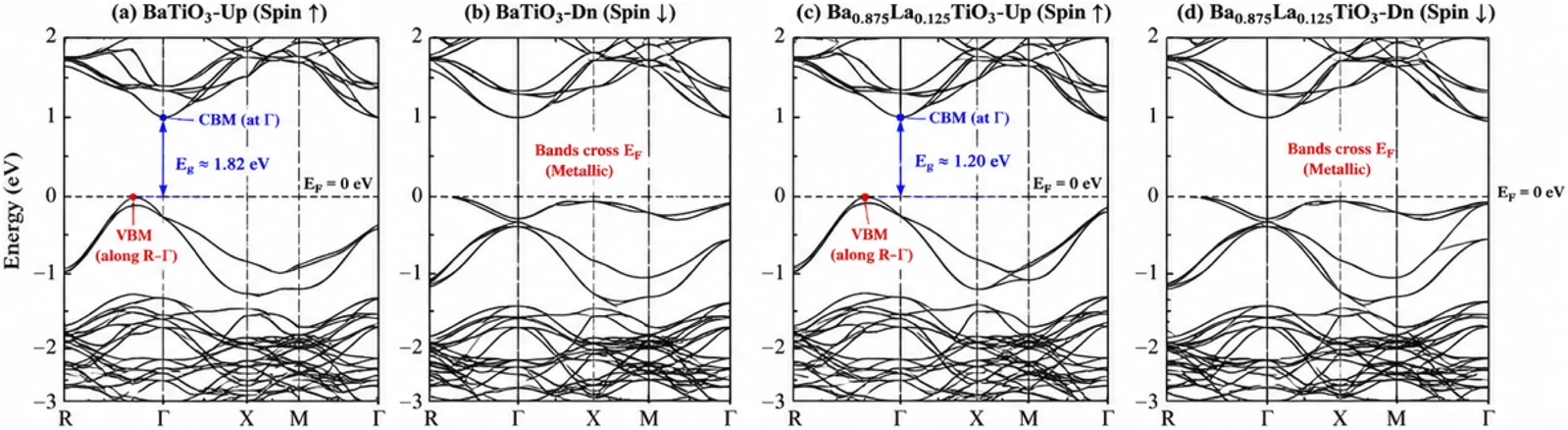
Spin-resolved electronic band structures of pristine BaTiO_3_ in the (a) spin-up and (b) spin-down channels, and Ba_0.875_La_0.125_TiO_3_ in the (c) spin-up and (d) spin-down channels. The Fermi level is set to 0 eV.

Upon La substitution at the Ba site (12.5% concentration), the overall topology of the band structure is preserved (see Fig. 3c and d).

The CBM remains located at the *Γ* point and retains its Ti-3d character, while the VBM remains largely O-2p–like and slightly off-Γ, consistent with the host material. Importantly, no localized impurity bands appear within the gap, reflecting the empty 4f shell of La^3+^ and its chemically benign role as a donor dopant. The absence of mid-gap states implies that La doping does not introduce deep traps or recombination centers, a favorable feature for dielectric and optoelectronic applications.

A key effect of La substitution is the upward shift of the Fermi level toward the conduction band. At the studied concentration, the Fermi level approaches or marginally enters the CBM, signaling the onset of degenerate *n*-type behavior. This shift occurs without significant distortion of the conduction-band dispersion, indicating that the Ti-3d transport backbone remains intact. The curvature of the lowest conduction band near *Γ* increases slightly relative to pristine BaTiO_3_, corresponding to a modest reduction in the electron effective mass and suggesting enhanced electron mobility under donor doping.

Because the electronic-structure calculations were performed using spin-polarized DFT, both spin-up and spin-down band structures are presented in Fig. 3. The two spin channels are nearly identical over the entire Brillouin zone, indicating negligible exchange splitting and confirming that substitutional La does not induce a magnetic ground state in BaTiO_3_ at the investigated concentration. The spin-resolved representation is included for completeness and demonstrates the absence of magnetically induced changes in the electronic structure.

#### 3.5.2. Density of states and donor-induced electronic screening

The total and projected density of states (DOS) further clarify the role of La substitution (see Fig. 4a and b). In pristine BaTiO_3_, the DOS near the band edges is dominated by O-2p states at the VBM and Ti-3d states at the CBM, with negligible Ba contribution. In La-doped BaTiO_3_, additional electronic states appear at the bottom of the conduction band, reflecting the donation of electrons by La^3+^; however, these states are fully delocalized and Ti-3d–like rather than dopant-localized.

**Fig. 4:**
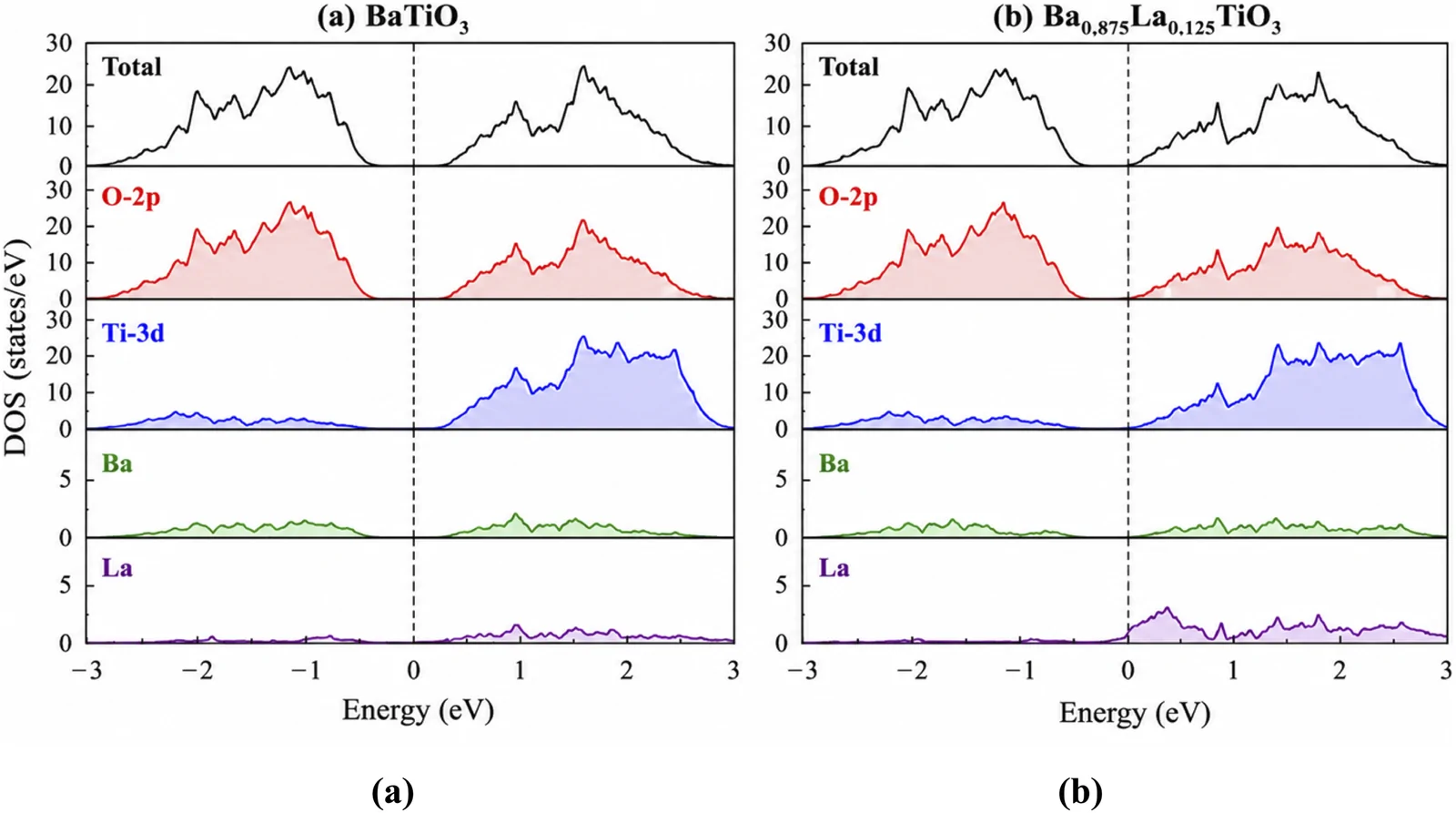
Total and orbital-resolved projected density of states (PDOS) of (a) pristine BaTiO_3_ and (b) Ba_0.875_La_0.125_TiO_3_. The valence-band maximum is dominated by O-2p states, whereas the conduction-band minimum is primarily composed of Ti-3d states. Ba and La states contribute only weakly near the band edges. The Fermi level is aligned at 0 eV.

Crucially, the DOS remains spin symmetric and free of sharp in-gap features, confirming the non-magnetic nature of La doping. The increase in electronic states near the CBM enhances low-energy electronic screening, which plays a central role in suppressing the ferroelectric instability and modifying the dielectric response, as discussed in Sections 3.3 and 3.4. At the same time, the preservation of a dispersive conduction band indicates that donor electrons contribute primarily to screening and transport rather than to localization or defect-assisted conduction.

#### 3.5.3. Burstein–Moss shift and band-edge renormalization

The upward movement of the Fermi level toward the bottom of the conduction band has direct consequences for the optical response. In the donor-shifted semiconducting regime considered here, partial occupation or near-edge filling of Ti-3d-derived conduction states can reduce the availability of low-energy optical transitions, producing a Burstein–Moss-like shift of the apparent absorption onset. As donor electrons occupy the lowest available conduction states, optical transitions from the valence band into these filled states are Pauli blocked. As a result, the apparent optical absorption edge shifts to higher photon energies a phenomenon known as the Burstein–Moss effect. This shift does not correspond to an intrinsic widening of the fundamental band gap but instead reflects a filling-induced renormalization of the optical threshold.

For La-doped BaTiO_3_ at 12.5% concentration, the calculated shift of the absorption onset is on the order of 0.1–0.2 eV relative to the undoped compound, consistent with experimental trends reported for donor-doped BaTiO_3_ ceramics and thin films. The magnitude of the shift correlates with the carrier concentration and the curvature of the conduction band, reinforcing the interpretation that the effect is driven by degenerate *n*-type doping rather than by changes in chemical bonding. The reported absorption-onset difference is interpreted subject to the electronic and optical limitations summarized in Section 2.1.

Fig. 5 shows optical absorption spectra illustrating the Burstein–Moss shift in pristine and La-doped BaTiO_3_. The absorption coefficient is plotted as a function of photon energy for pristine BaTiO_3_ and Ba_0.875_La_0.125_TiO_3_. La substitution shifts the apparent optical absorption edge toward higher energies due to donor-induced filling of the conduction-band minimum, which blocks low-energy interband transitions. The shaded region highlights the Burstein–Moss shift, while the dashed lines indicate the effective optical band edges before and after doping. The upward shift occurs without a fundamental widening of the intrinsic band gap, confirming that the effect originates from carrier occupation rather than changes in chemical bonding.

**Fig. 5:**
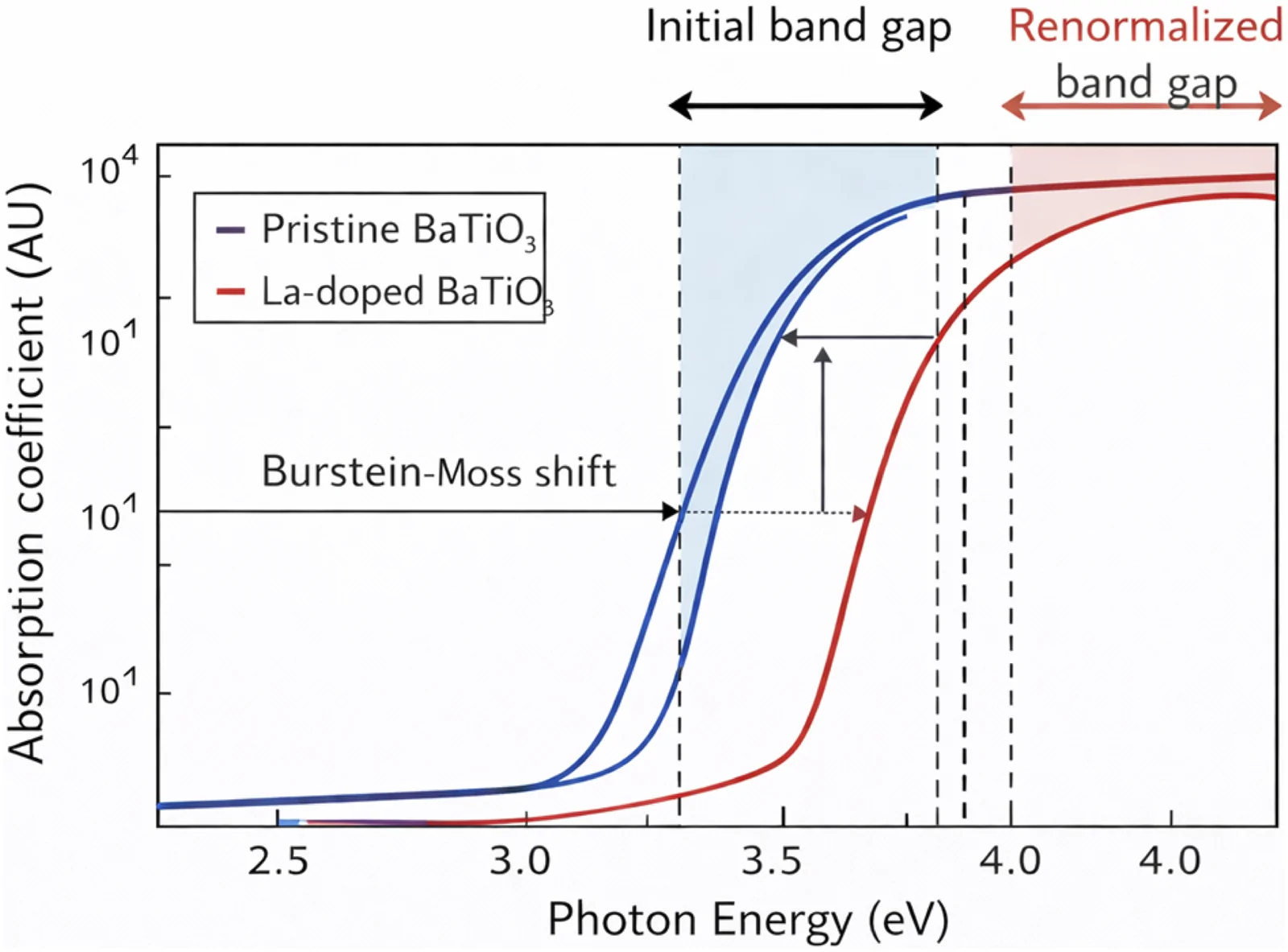
Optical absorption spectra of pristine BaTiO_3_ and Ba_0.875_La_0.125_TiO_3_ illustrating the Burstein–Moss-like shift of the absorption onset. The dashed vertical lines mark the effective optical absorption edges, while the shaded region highlights the donor-induced shift after La substitution. The shift originates from near-CBM band filling and Pauli blocking of low-energy interband transitions rather than from a fundamental widening of the intrinsic band gap.

### 3.6. Optical properties

#### 3.6.1. Complex dielectric function *ε*_1_(*ω*) and *ε*_2_(*ω*)

The optical response of La-substituted BaTiO_3_ was evaluated through the complex dielectric function, *ε*(*ω*) = *ε*_1_(*ω*) + i*ε*_2_(*ω*), derived from the momentum matrix elements of interband electronic transitions within the independent-particle approximation.

For pristine BaTiO_3_, the imaginary component *ε*_2_(*ω*) (see Fig. 6a) displays a distinct threshold at the fundamental optical gap, followed by a strong peak in the ultraviolet region at approximately 4.5–5.0 eV. This feature originates predominantly from transitions between O 2p-derived valence states and Ti 3d conduction states, which define the fundamental electronic excitation channel of the perovskite framework. At higher photon energies, *ε*_2_(*ω*) exhibits broader structures extending up to ∼10 eV, reflecting additional interband contributions within the conduction manifold. The overall spectral features are governed by O-2p → Ti-3d transitions; therefore, while the absolute photon energies are slightly underestimated within semilocal DFT, the relative spectral redistribution and anisotropy trends remain robust.

**Fig. 6:**
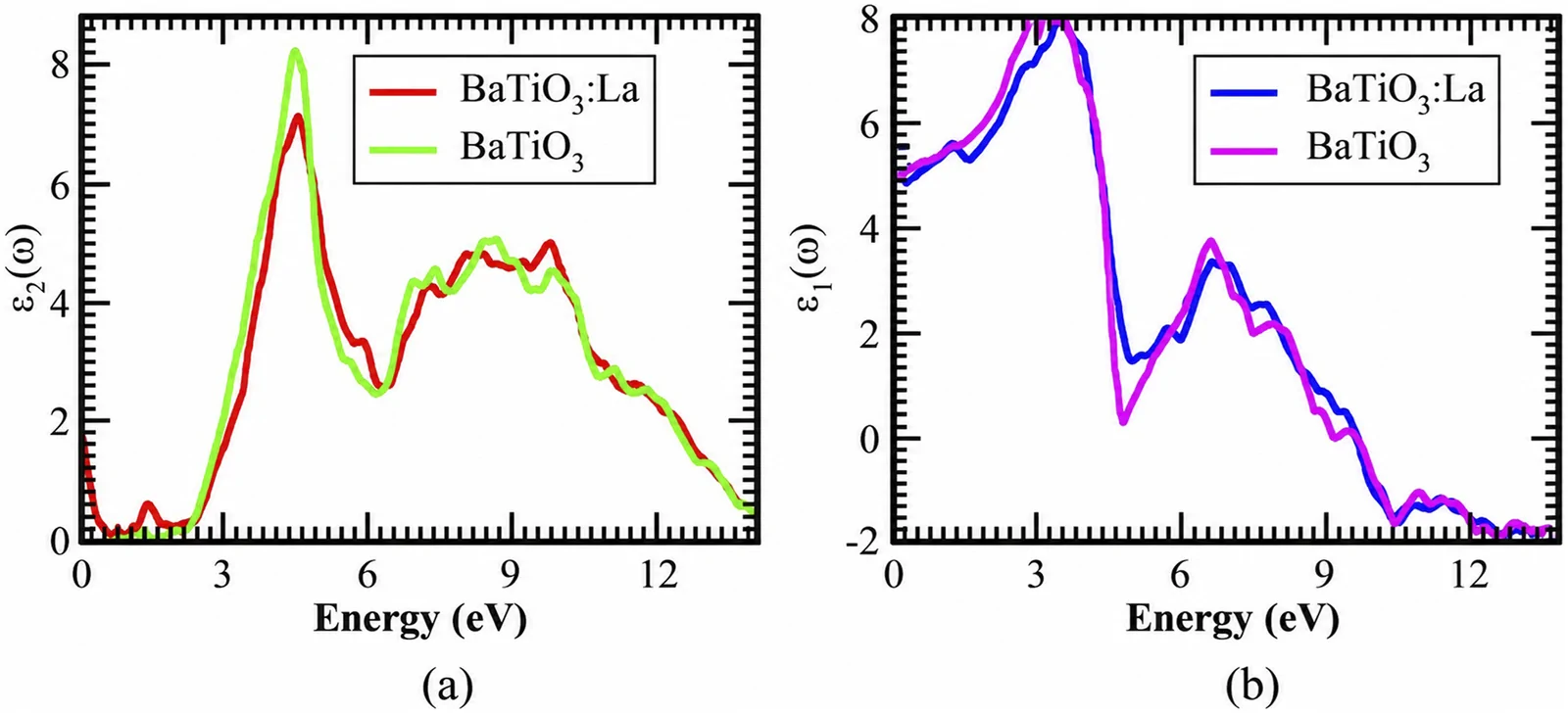
Real and imaginary parts of the dielectric function, *ε*_1_(*ω*) and *ε*_2_(*ω*), for pristine and La-doped BaTiO_3_. The same photon-energy range, axis scale, font style, and color scheme are used for both spectra to enable direct comparison of doping-induced optical changes.

For pristine BaTiO_3_, The real part of the dielectric function (see Fig. 6b) shows smooth dispersion below the absorption edge, with a low-energy electronic dielectric response of approximately 5.3–5.6, consistent with the range reported in Section 3.4.1.

Near the principal interband transition, *ε*_1_(*ω*) undergoes a pronounced reduction, reflecting the strong coupling between incident photons and the Ti–O electronic network.

La substitution preserves the overall spectral topology of both *ε*_1_(*ω*) and *ε*_2_(*ω*), confirming that the dominant Ti–O hybridized electronic structure remains intact. However, two systematic trends emerge. First, the onset of *ε*_2_(*ω*) shifts toward slightly higher photon energies, consistent with a Burstein–Moss-type effect arising from donor electrons introduced by La^3+^ substitution. The upward shift of the Fermi level reduces the number of available low-energy transitions, effectively widening the apparent optical gap. Second, *ε*_1_(*ω*) exhibits a modest enhancement in the low-energy regime, indicating strengthened electronic screening due to increased carrier density. Importantly, no additional sub-gap absorption features are observed, demonstrating that La incorporation does not generate optically active mid-gap defect states within the calculated energy window.

#### 3.6.2. Absorption edge shift and optical transparency

The absorption coefficient, extracted from *ε*_2_(*ω*), further clarifies the impact of La substitution on optical excitation processes (see Fig. 7). In pristine BaTiO_3_, absorption rises sharply at the band edge and reaches substantial values in the ultraviolet region, consistent with strong O 2p → Ti 3d interband transitions.

**Fig. 7:**
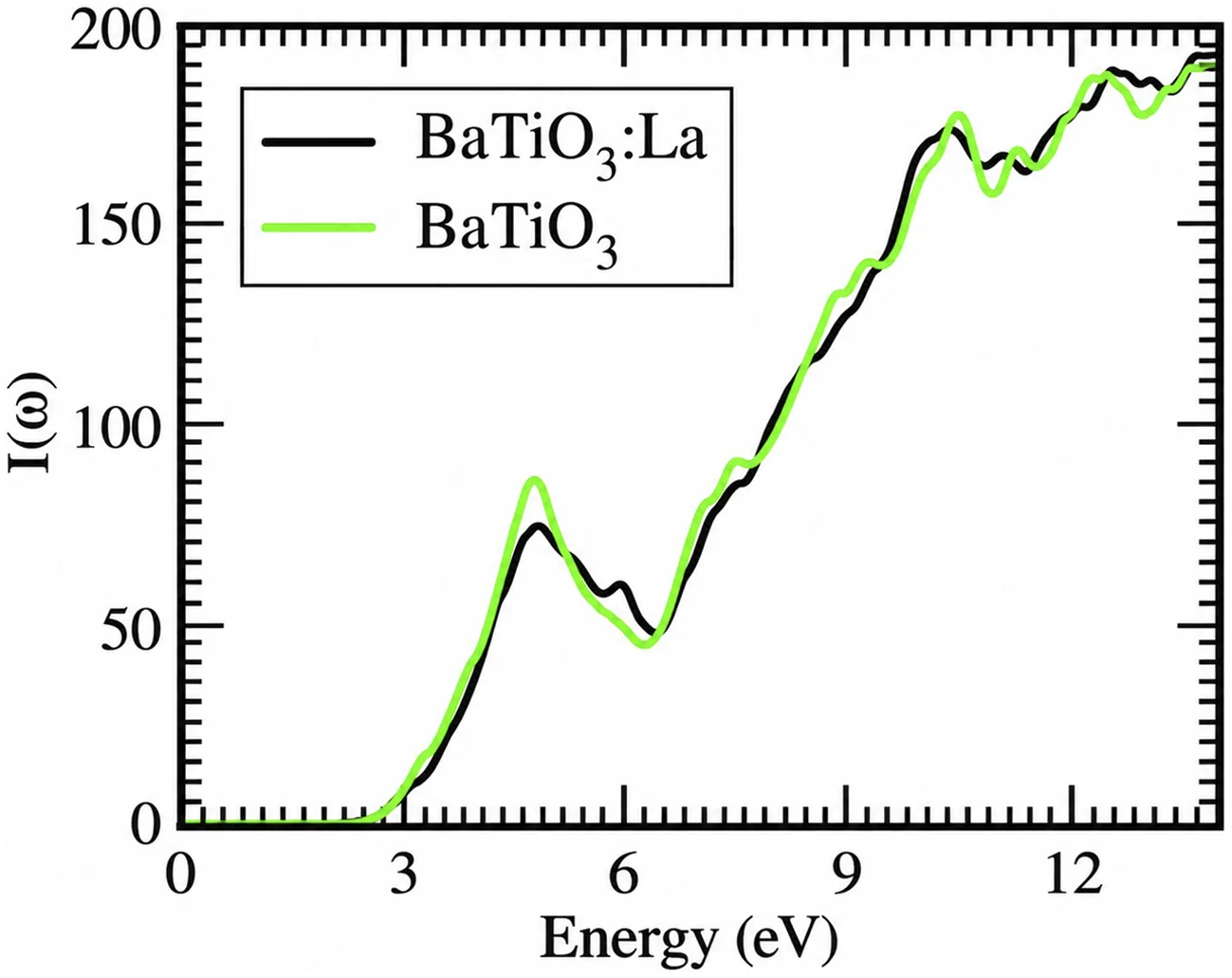
Absorption coefficient, *I*(*ω*), for pristine and La-doped BaTiO_3_. The absorption edge shifts slightly to higher energy upon La incorporation, while UV absorption remains strong.

Upon La doping, the effective absorption edge shifts toward higher photon energies, in agreement with the dielectric-function analysis and the predicted Burstein–Moss mechanism. Despite this shift, the absorption spectra of pristine and La-doped BaTiO_3_ nearly coincide above ∼4 eV, indicating that high-energy optical transitions remain governed by the intrinsic Ti–O electronic framework rather than dopant-induced states.

This behavior has important implications. The upward shift of the absorption edge enhances transparency in the near-UV and visible regions while preserving strong ultraviolet absorption. In practical terms, La substitution enables tuning of optical transparency without sacrificing the intrinsic UV optical activity that is characteristic of BaTiO_3_-based dielectric and optoelectronic materials. The optical spectra should be interpreted within the approximations described in Section 2.1.

#### 3.6.3. Refractive index and birefringence reduction

The refractive index *n* (*ω*), obtained from *ε*_1_(*ω*) and *ε*_2_(*ω*), exhibits a maximum near the fundamental absorption edge and gradually decreases at higher photon energies due to normal dispersion (see Fig. 8a). For pristine BaTiO_3_, the static electronic refractive index (n as *ω* → 0) lies in the range 2.45–2.50, consistent with experimental reports and previous first-principles calculations.

**Fig. 8:**
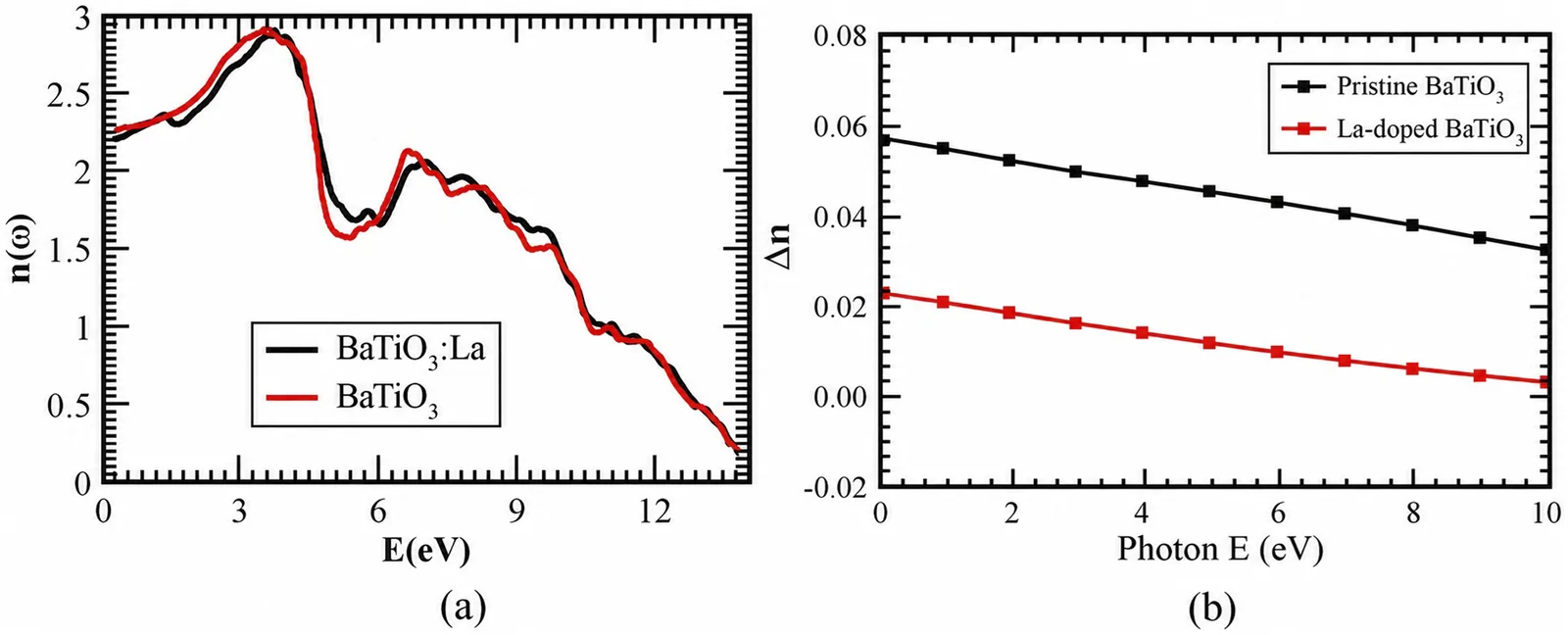
Refractive index, *n*(*ω*), and birefringence, Δ*n*(*ω*), of pristine and La-doped BaTiO_3_. Unified formatting, identical photon-energy scale, and consistent curve styles are used to highlight the reduction of optical anisotropy upon La substitution.

Upon La substitution, a slight increase in the low-energy refractive index is observed, consistent with the modest enhancement of *ε*_∞_. Beyond the absorption edge, however, the overall dispersion profile remains largely unchanged, indicating that the dominant Ti–O optical framework is preserved after La incorporation.

In tetragonal BaTiO_3_, optical anisotropy gives rise to birefringence, defined as Δ*n* = *n*_e_ – *n*_o_, where *n*_e_ and *n*_o_ denote the extraordinary and ordinary refractive indices, respectively, as shown in Fig. 8b. For pristine BaTiO_3_, Δ*n* is approximately 0.05–0.06 in the visible region, reflecting the intrinsic tetragonal and polar anisotropy of the ferroelectric phase. After La substitution, Δ*n* decreases to approximately 0.03–0.04. This reduction is consistent with the weakened lattice and polarization anisotropy discussed in the preceding structural and ferroelectric sections.

The finite calculated birefringence indicates that the La-substituted model retains optical anisotropy.

Overall, La substitution preserves the main Ti–O-derived optical response while moderately tuning the refractive index and reducing birefringence. These results suggest that A-site donor substitution can adjust optical anisotropy in BaTiO_3_ without introducing additional sub-gap optical features or significantly disrupting the intrinsic ultraviolet optical activity.

#### 3.6.4. Unified structure–polarization–optical correlation

The reported reduction in birefringence accompanies decreases in Ti off-centering, selected anomalous Born effective charges, and Berry-phase polarization. These observations motivate further examination of a possible screening contribution, but the increase in *c*/*a* derived from Table 3 prevents interpreting them as evidence of reduced tetragonality. The optical analysis presented here is distinguished from conventional optical studies of doped BaTiO_3_ in that it does not treat the dielectric function, absorption edge, refractive index, and birefringence as isolated spectral quantities. Instead, these optical parameters are interpreted in direct connection with the calculated structural, ferroelectric, and dielectric descriptors. The Burstein–Moss-type interpretation of the absorption-edge shift is based on the reported electronic occupations and Fermi-level movement. Separately, reduced birefringence accompanies smaller Ti off-centering, selected Born effective charges, and spontaneous polarization. These accompanying changes do not establish a quantitative causal relationship, and the listed lattice constants do not support linking the optical change to decreased tetragonality.

The reported optical and polar changes suggest a possible connection that requires further investigation. However, the present comparison does not establish a quantitative or causal relationship among tetragonality, polarization, and birefringence. In particular, the lattice constants indicate increased *c*/*a* despite the reported decreases in polarization and birefringence.

The reported Ti off-centering decreases from approximately 0.13 Å in pristine BaTiO_3_ to 0.09 Å in the La-substituted model. This internal displacement change must be distinguished from the axial distortion: the lattice constants in Table 3 give *c*/*a* ≈ 1.0100 and 1.0126, respectively, indicating a slight increase in tetragonality. Smaller polar displacements can contribute to reduced spontaneous polarization, but the reported polarization change cannot be explained by a decrease in *c*/*a*.

Second, donor-induced screening simultaneously reduces the anomalous Born effective charges of Ti and O. The Ti dynamical charge decreases from approximately +7.1 to +6.7, reflecting weakened Ti–O hybridization and diminished dynamic charge transfer. Because spontaneous polarization can be expressed schematically as:
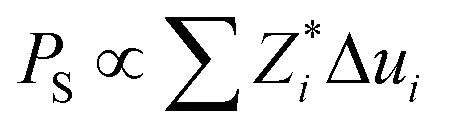


the combined reduction of both Z* and Δ*u* produces a decrease in Berry-phase polarization from ∼26 µC cm^−2^ to ∼21 µC cm^−2^.

Optical birefringence reflects anisotropy in the frequency-dependent electronic response. The reported birefringence decreases from Δ*n* ≈ 0.055 in pristine BaTiO_3_ to approximately 0.035 upon La substitution, alongside a decrease in spontaneous polarization. However, the lattice constants in Table 3 indicate a slight increase in *c*/*a*. The decrease in birefringence therefore cannot be attributed to reduced tetragonality on the basis of these structural values. This two-composition comparison also does not establish a quantitative scaling relationship between birefringence, polarization, and axial distortion.

The reported results show polar-mode hardening, smaller Ti off-centering, reduced selected anomalous Born effective charges, lower spontaneous polarization, and decreased birefringence upon La substitution. Enhanced electronic screening is a possible contribution to these changes, but the present comparison does not isolate its effect from changes in local bonding and internal geometry. Furthermore, the reported lattice constants indicate increased *c*/*a*. The results therefore do not establish a mechanism in which decreased tetragonality connects all of the reported functional changes.

Using the approximate values quoted above, birefringence decreases by about 36%, while spontaneous polarization decreases by about 19%. These percentage changes describe the comparison between the two compositions; they do not establish a linear, nonlinear, or monotonic scaling law.

The trends identified for La-doped BaTiO_3_ should be interpreted as representative of a broader donor-screening mechanism rather than as effects unique only to La. For other A-site donor dopants, such as Ce, Nd, Sm, or Gd, the same basic physical pathway may operate: donor substitution increases electronic screening, weakens the ferroelectric soft mode, reduces Ti off-centering, suppresses anomalous Born effective charges, and consequently lowers polarization and optical birefringence. Nevertheless, dopant-specific differences are expected. Ce, for example, can exhibit mixed valence behavior depending on synthesis conditions and charge compensation, while some rare-earth dopants contain localized 4f electrons that may introduce additional electronic, magnetic, or optical features. Similarly, donor dopants with different ionic radii may impose different levels of chemical pressure and local strain. Therefore, La doping provides a useful reference case for isolating the general donor-screening effect, whereas comparison with Ce or other rare-earth dopants would be valuable for separating universal donor-doping behavior from dopant-specific electronic-structure effects.

## 4. Conclusion

In this work, first-principles calculations were employed to examine how La donor substitution modifies the structural, ferroelectric, dielectric, electronic, and optical properties of BaTiO_3_. The reported lattice constants indicate volume contraction and a slight increase in tetragonality upon La incorporation. Separately, the reported phase energies indicate reduced stabilization of the tetragonal structure relative to the cubic reference, while the phonon dispersions indicate that the doped structure remains dynamically stable. The upward shift of the low-frequency polar mode further indicates a partial suppression of the ferroelectric soft-mode instability without structural collapse. The reported ferroelectric and dielectric changes accompany reduced Ti off-centering and a smaller tetragonal stabilization energy, despite the slight increase in *c*/*a* derived from the listed lattice constants. Berry-phase calculations show a reduction in spontaneous polarization, mainly associated with reduced Ti off-centering and weakened anomalous Born effective charges. Consistently, the dielectric response changes from a strongly lattice-dominated behaviour toward a more screening-influenced regime, with a modest increase in the electronic contribution and a reduction in the lattice-mediated component. The electronic and optical results support the same physical picture. La substitution preserves the intrinsic Ti–O band topology and does not introduce mid-gap defect states, indicating that the donor effect is mainly associated with near-band-edge electronic filling rather than trap-state formation. The upward movement of the Fermi level toward the conduction-band minimum is interpreted as contributing to a Burstein–Moss-like shift of the optical absorption edge. The reported reduction in birefringence accompanies lower spontaneous polarization, but it cannot be attributed to decreased tetragonality because the listed lattice constants yield a slight increase in *c*/*a*. The study compares structural, energetic, ferroelectric, dielectric, and optical properties for pristine BaTiO_3_ and one La-substituted configuration. The reported functional changes motivate an interpretation involving enhanced electronic screening, but the present comparison does not establish a unique causal mechanism. In particular, the lattice parameters indicate a slight increase in tetragonality, so decreased *c*/*a* cannot serve as the structural explanation for the reported polarization and birefringence reductions. These findings should be interpreted within the scope and validity conditions summarized in Section 2.1. Overall, the Ba_0.875_La_0.125_TiO_3_ model provides a coherent picture of how A-site donor substitution can tune the coupled ferroelectric, dielectric, and optical response of BaTiO_3_. Future studies involving multiple La concentrations, different dopant configurations, explicit charge-compensation defects, and higher-level optical treatments would be valuable for establishing a quantitative doping–property relationship in donor-engineered ferroelectric oxides.

## Conflicts of interest

There are no conflicts to declare.

## Data Availability

The data supporting the findings of this study are available within the article. Additional data generated during the first-principles calculations are available from the corresponding author upon reasonable request.
